# Generation of iPSCs by Nonintegrative RNA-Based Reprogramming Techniques: Benefits of Self-Replicating RNA versus Synthetic mRNA

**DOI:** 10.1155/2019/7641767

**Published:** 2019-06-19

**Authors:** Heidrun Steinle, Marbod Weber, Andreas Behring, Ulrike Mau-Holzmann, Christian Schlensak, Hans Peter Wendel, Meltem Avci-Adali

**Affiliations:** ^1^University Hospital Tübingen, Department of Thoracic and Cardiovascular Surgery, Calwerstraße 7/1, 72076 Tübingen, Germany; ^2^University Hospital Tübingen, Institute of Medical Genetics and Applied Genomics, Calwerstraße 7, 72076 Tübingen, Germany

## Abstract

The reprogramming of somatic cells into induced pluripotent stem cells (iPSCs) is gaining in importance in the fields of regenerative medicine, tissue engineering, and disease modeling. Patient-specific iPSCs have as an unlimited cell source a tremendous potential for generating various types of autologous cells. For the future clinical applicability of these iPSC-derived cells, the generation of iPSCs via nongenome integrating methods and the efficient reprogramming of patients' somatic cells are required. In this study, 2 different RNA-based footprint-free methods for the generation of iPSCs were compared: the use of synthetic modified messenger RNAs (mRNAs) or self-replicating RNAs (srRNAs) encoding the reprogramming factors and GFP. Using both RNA-based methods, integration-free iPSCs without genomic alterations were obtained. The pluripotency characteristics identified by specific marker detection and the in vitro and in vivo trilineage differentiation capacity were comparable. Moreover, the incorporation of a GFP encoding sequence into the srRNA enabled a direct and convenient monitoring of the reprogramming procedure and the successful detection of srRNA translation in the transfected cells. Nevertheless, the use of a single srRNA to induce pluripotency was less time consuming, faster, and more efficient than the daily transfection of cells with synthetic mRNAs. Therefore, we believe that the srRNA-based approach might be more appropriate and efficient for the reprogramming of different types of somatic cells for clinical applications.

## 1. Introduction

The reprogramming of a patient's somatic cells into induced pluripotent stem cells (iPSCs) is mediated by the exogenous delivery of the “Yamanaka” factors Oct4, Klf4, Sox2, and cMyc, and it allows the generation of an unlimited stem cell source for tissue regeneration [1–3]. In the first studies, retroviral vectors were used to deliver the reprogramming factors into cells. However, the therapeutic application of cells derived from these iPSCs is hampered due to the risks associated with the random integration of viral vectors into the host genome.

In recent years, various nonintegrative reprogramming methods have been successfully established to induce pluripotency in different somatic cell types [4–8]. One of the most promising approaches is the use of a synthetic modified mRNA for reprogramming [6, 9–11]. After the delivery of synthetic mRNA into the cytosol, the mRNA is immediately translated by ribosomes into proteins and the entry into the nucleus is not required. The synthesis of reprogramming factors ceases after the degradation of mRNA, and no footprints are left. Furthermore, during the in vitro transcription (IVT), the synthetic mRNA can be modified with a cap structure, poly(A) tail, and modified nucleosides to improve the stability and the translation of proteins [12–17]. Previous studies showed that modified nucleosides, e.g., pseudouridine (Pseudo-UTP) and 5-methylcytidine (5mCTP), can be incorporated into the synthetic mRNA to substitute cytidine and uridine to abrogate the innate immune response. However, despite the great advances in the development of synthetic mRNA-based reprogramming approaches, one of the main obstacles is still the induction of an innate immune response following multiple daily mRNA transfections, resulting in increased cellular stress and severe cytotoxicity [18, 19]. Thus, to prevent interferon-response induced cell death, the reprogramming medium needs to contain the interferon inhibitor B18R derived from vaccinia virus [6, 20, 21].

Another alternative to synthetic mRNA-based reprogramming is the use of self-replicating RNA (srRNA) [22]. The srRNA contains the coding sequences of the “Yamanaka” transcription factors Oct4, Klf4, Sox2, and cMyc and four nonstructural proteins (nsP1 to nsP4), which encode the RNA replication complex of Venezuelan equine encephalitis (VEE) virus [22–24]. The srRNA is a single-stranded RNA that mimics cellular 5′-capped and 3′-polyadenylated mRNA. The application of srRNA enables an extended duration of protein expression. To date, no risk for genomic integration has been reported by the generation of DNA intermediates [23, 25]. However, the presence of B18R protein is also required during the srRNA-based reprogramming as in synthetic mRNA-based reprogramming.

In this work, we compared the synthetic mRNA- and srRNA-based reprogramming methods to generate iPSCs from human neonatal fibroblasts. The one-time delivery of 1 *μ*g srRNA significantly improved the reprogramming efficiency of fibroblasts compared to the daily transfection of cells with 1.2 *μ*g mRNAs for at least 2 weeks. The srRNA-based reprogramming enhanced the reprogramming of somatic cells and resulted in increased numbers of iPSCs compared to synthetic mRNA-based reprogramming. Furthermore, the incorporation of the GFP encoding sequence to the srRNA enabled the monitoring of the reprogramming procedure and optimization of the culture conditions.

## 2. Materials and Methods

### 2.1. mRNA Synthesis

The pcDNA 3.3 plasmids containing the coding sequence for either Klf4, cMyc, Oct4, Sox2, Lin28, or eGFP [6] were purchased from Addgene (LGC Standards, Teddington, UK). DNA templates for the in vitro transcription (IVT) of mRNAs were generated with a polyT_120_ sequence. Subsequently, the mRNA synthesis and modifications were performed according to the previously published methods by Avci-Adali et al. [26, 27]. Briefly, to generate DNA templates, PCR was performed using 50-100 ng plasmid DNA and a forward primer (5′-TTGGACCCTCGTACAGAAGCTAATACG-3′) and reverse primer (5′-T_120_CTTCCTACTCAGGCTTTATTCAAAGACCA-3′). During the IVT reaction, 1.5 *μ*g DNA, ATP, GTP, pseudoruridine-5′-triphosphate (Pseudo-UTP), 5-methylcytidine-5′-triphosphate (5mCTP), and 3′-0-Me-m7G(5′)ppp(5′)G RNA Cap Structure Analog was used. The incubation was performed at 37°C for 4 h. After dephosphorylation, the mRNA was purified and the concentration was adjusted to 100 ng/*μ*l in nuclease-free water. Subsequently, produced mRNAs were analyzed using 1% agarose gel electrophoresis and gels were stained with GelRed™ in 1x TBE buffer.

### 2.2. srRNA Synthesis

**Figure 1:**
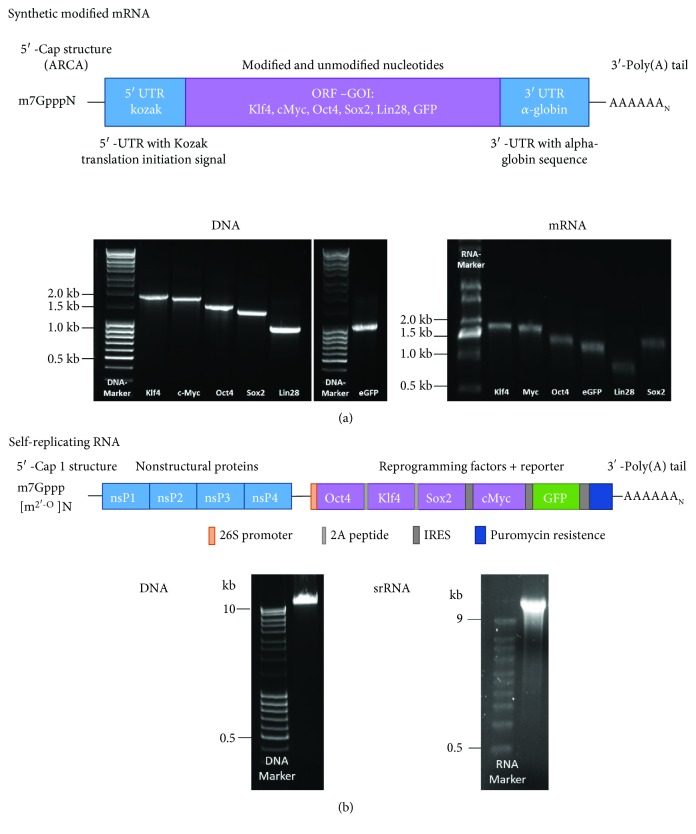
Schematic representation of RNA constructs and quality control of DNA templates and synthesized RNA. The srRNA and synthetic modified mRNAs were synthesized using in vitro transcription (IVT). (a) Agarose gel electrophoresis showed the expected lengths for mRNAs encoding the reprogramming factors (Klf4: 1.6 kb; cMyc: 1.5 kb; Oct4: 1.3 kb; Sox2: 1.1 kb; and Lin28: 0.8 kb) and eGFP (0.9 kb). (b) The srRNA contains encoding sequences for the nonstructural proteins (nsP1-4); the reprogramming factors Oct4, Klf4, Sox2, and cMyc; and GFP. Agarose gel electrophoresis showed the expected length (17.7 kb) of linearized DNA and srRNA.

The T7-VEE-OKS-iM plasmids containing the coding sequences for Oct4, Sox2, Klf4, and cMyc [22] were purchased from Addgene (LGC Standards). For monitoring the transfection and reprogramming efficiency, an IRES (internal ribosome entry site)-GFP reporter encoding sequence was inserted by Aldevron (Fargo, USA) into the plasmid (Figure 1(b)). Thereby, the T7-VEE-OKS-iMG plasmid was obtained. To multiply the T7-VEE-OKS-iMG plasmid, competent *E. coli* cells (*α*-select chemically competent cells from Bioline GmbH, Luckenwalde, Germany) were transformed with 100 ng plasmid DNA and cultivated in LB medium supplemented with 50 *μ*g/ml ampicillin (Sigma-Aldrich Chemie GmbH, Steinheim, Germany). The isolation of plasmids was performed using the QIAprep Spin Miniprep Kit (Qiagen). Linearized DNA templates were generated using the FastDigest MluI restriction enzyme (Thermo Fisher Scientific). Therefore, 36 *μ*g plasmid was incubated for 3 h at 37°C with 5 U enzyme, 20 *μ*l 1x reaction buffer, and nuclease-free water in a total volume of 200 *μ*l. Afterwards, linearized DNA was purified using the ISOLATE II PCR and Gel Kit (Bioline) and the complete linearization, purity, and specific length were analyzed by 1% agarose gel electrophoresis. Next, IVT was performed using the RiboMAX Large-Scale Production System T7 Kit (Promega, Madison, USA) according to the manufacturer's instructions. The IVT reaction was prepared with 10 *μ*g template DNA and contained 40 U of the RNAse Inhibitor (Thermo Fisher Scientific) to prevent the degradation of srRNA. Afterwards, 5′-end capping (Cap1) was performed using the ScriptCap Cap1 Capping System followed by 3′-end polyadenylation with the A-Plus Poly(A) Polymerase Tailing Kit (both from CELLSCRIPT, Madison, USA) according to the manufacturer's instructions. Following each reaction step, the srRNA was purified using the ISOLATE II RNA Mini Kit (Bioline). The specific length and purity of the generated srRNA products was analyzed by 1% agarose gel electrophoresis containing 2.2 M formaldehyde in 1x MOPS (3-(N-morpholino)propanesulfonic acid) buffer at 100 V for 60 min. The gels were stained using 1x GelRed™ (Biotium, Fremont, USA) in 1x MOPS buffer.

### 2.3. Cultivation of Fibroblasts

Neonatal human foreskin fibroblasts (NuFFs, untreated, passage 9, Amsbio, Milton Park, UK) were cultivated in DMEM high glucose supplemented with 10% FBS, 1x GlutaMAX, 10 mM HEPES, and 50 *μ*g/ml gentamicin B. These cell culture reagents were obtained from Thermo Fisher Scientific. Cells were cultivated at 37°C with 5% CO_2_ (normoxia), and medium was changed every 2-3 days. Cells were detached at about 70% confluency using 0.04% trypsin/0.03% EDTA, and then trypsin neutralizing solution (TNS, 0.05% trypsin inhibitor in 0.1% BSA, PromoCell, Heidelberg, Germany) was added. Afterwards, cells were centrifuged for 5 min at 300 x g, resuspended in culture medium, and seeded at the desired cell density for reprogramming.

To generate inactivated feeder cells, NuFFs and mouse embryonic fibroblasts (MEFs, CF-1, untreated, passage 3, Amsbio) were treated with 10 mg/ml mitomycin C (Merck, Darmstadt, Germany) and frozen in 10% DMSO containing cell culture medium. The wells of 6-well plates were coated with 0.1% gelatin (Sigma-Aldrich Chemie GmbH) in fibroblast culture medium for 4 h at 37°C. For the mRNA-based reprogramming 2.5 × 10^5^ inactivated NuFFs or for the cultivation of primarily picked mRNA-iPSCs 1.5 × 10^5^ inactivated MEFs were seeded per well of a 0.1% gelatin coated 6-well plate and cultivated overnight.

### 2.4. Reprogramming of Cells Using Synthetic mRNA

To perform synthetic mRNA-mediated reprogramming, 2 × 10^4^ fibroblasts were seeded per well of a 6-well plate, which was preseeded with 2.5 × 10^5^ inactivated NuFF feeder cells in cultivation medium. The next day, Pluriton reprogramming medium (Stemgent, Cambridge, USA) was equilibrated at hypoxia (5% O_2_ and 5% CO_2_, at 37°C) for 2 h and supplemented with 200 ng/ml B18R interferon inhibitor protein (Thermo Fisher Scientific) to repress synthetic mRNA-mediated immune activation. Then, the mRNA transfection cocktail was prepared with a molar ratio of 3 : 1 : 1 : 1 : 1 : 1 for Oct4, Klf4, cMyc, Sox2, Lin28, and eGFP mRNA, respectively. For each transfection, 1.2 *μ*g (100 ng/*μ*l) mRNA cocktail and 4 *μ*l Lipofectamine 2000 (Thermo Fisher Scientific) were incubated in 120 *μ*l Opti-MEM™ I Reduced Serum Medium (Opti-MEM, Thermo Fisher Scientific) for 15 min at room temperature (RT) to form lipoplexes. The transfection complexes were then added dropwise to the cells and incubated for 4 h at hypoxia. Afterwards, the complexes were aspirated and 2 ml B18R containing Pluriton medium was added to the transfected fibroblasts and incubated for 24 h at hypoxia. The transfection of cells was performed daily for 20 days. At day 6, the medium was changed to NuFF-conditioned Pluriton medium. NuFF-conditioned medium was obtained by seeding 4 × 10^6^ inactivated NuFFs in T75 cell culture flasks and incubating cells with 25 ml of Pluriton medium supplemented with 4 ng/ml bFGF (PeproTech, Hamburg, Germany). The medium was collected 6x after overnight incubation, pooled, and sterile-filtered using a 0.2 *μ*m filter.

### 2.5. Reprogramming of Cells Using srRNA

To reprogram NuFFs using synthetic srRNAs, 5 × 10^4^ NuFFs (passage 12) were seeded per well of a 6-well plate coated with 0.1% gelatin and incubated overnight at 37°C in fibroblast culture medium. The next day, cells were incubated at hypoxia with B18R-conditioned Pluriton medium (BcM) for 45-60 min. To generate BcM, 2 × 10^6^ NuFFs were seeded in a T75 cell culture flask and cultivated overnight to reach a confluency of 70%. Then, cells were transfected with 7.5 *μ*g B18R mRNA, 15 *μ*l Lipofectamine 2000, and 7 ml Opti-MEM for 4 h at 37°C. Afterwards, the mRNA complexes were aspirated from the cells and 15 ml fibroblast culture medium was added and incubated overnight. The BcM was collected 3x after overnight incubation, pooled, and sterile-filtered using a 0.2 *μ*m filter. Before application, the collected medium (100% BcM) was diluted 1 : 4 with either Pluriton or E8 medium (resulting in 25% BcM). For the transfection of cells, lipoplexes were generated by the incubation of 1 *μ*g srRNA for 15 min at RT with 3 *μ*l Lipofectamine MessengerMAX (Thermo Fisher Scientific) in 1 ml Opti-MEM. Then, medium was aspirated and Opti-MEM containing lipoplexes were added to the cells. After 4 h of incubation, the transfection medium was discarded and 2 ml Pluriton medium containing 25% BcM was added for further incubation at hypoxia for 24 h. The next day, medium was replaced by 2 ml Pluriton medium containing 25% BcM. To select the srRNA-transfected cells 1 or 2 days posttransfection, when the cells reached confluency, 0.8 *μ*g/ml puromycin (Sigma-Aldrich Chemie GmbH) was added to the medium to eliminate srRNA negative cells. After 2-3 days of puromycin treatment, Pluriton medium containing 25% BcM was changed every 1-2 days, depending on cell density and viability. At day 7, the medium was changed to a stem cell medium (E8) containing 25% BcM/E8. After the first iPSC colonies appeared (days 12-14), the E8 medium without BcM (B18R) was used.

### 2.6. Cultivation of iPSCs

Primary iPSC colonies were stained with mouse anti-human StainAlive™ SSEA-4 DyLight™ 550 antibody (Stemgent). iPSCs obtained by the synthetic mRNA delivery were picked manually and transferred into one well of a 12-well plate preseeded with 1.5 × 10^5^ iMEF feeder cells. The cultivation was performed in a standard stem cell medium containing DMEM/F12 high glucose supplemented with 20% KnockOut™ Serum Replacement, 1x GlutaMAX, 1x minimum essential medium (MEM) nonessential amino acids (NEAA), 20 ng/ml bFGF (PeproTech), 100 *μ*M 2-mercaptoethanol, and 1x penicillin/streptomycin. After 2-3 passages, iPSCs were adapted to feeder-free conditions by cultivation on surfaces coated with 0.5 mg/cm^2^ truncated recombinant human vitronectin (VTN-N) in Essential 8 (E8) medium. Unless otherwise indicated, all cell culture reagents were obtained from Thermo Fisher Scientific. iPSCs obtained by the srRNA delivery were also picked manually and cultivated on 0.5 mg/cm^2^ VTN-N coated 12-well plates in E8 medium. Passaging of iPSCs was performed every 5-7 days at a split ratio of 1 : 10 with 0.5 mM EDTA in Dulbecco's phosphate-buffered saline (DPBS) for 5-10 min at RT. After the aspiration of ETDA solution, colonies were detached using the E8 medium supplemented with a 10 *μ*M Y-27632 ROCK inhibitor (Enzo Life Science, Lörrach, Germany) to increase single cell survival.

### 2.7. Immunocytochemistry

iPSCs (passage 4 to 7) were seeded onto VTN-N-coated glass slides in 12-well plates and cultivated at normoxia until reaching confluency of about 60% in E8 medium. Then, the cells were washed twice with 1 ml DPBS and fixated with 500 *μ*l fixation solution (R&D Systems, Minneapolis, USA) for 15 min at RT. Next, cells were washed again 2 times with 1 ml DPBS and blocked with 500 *μ*l 5% BSA in wash buffer (Permeabilization/Wash Buffer I, R&D Systems) for 2 h at RT or overnight at 4°C. Primary antibodies were incubated overnight at 4°C in 500 *μ*l 1% BSA in wash buffer, according to the manufacturer's instructions. The following unlabelled and fluorescently labelled primary antibodies were used: rabbit anti-human POU5F1 (Oct4) antibody (Sigma-Aldrich Chemie GmbH), rabbit anti-mouse/human Sox2 antibody (Stemgent), mouse anti-human LIN28A monoclonal antibody (6D1F9) (Thermo Fisher Scientific), mouse anti-human PE Nanog antibody (BD Biosciences, Franklin Lakes, USA), mouse anti-human StainAlive™ TRA-1-60 antibody (DyLight™ 488) (Stemgent), and mouse anti-human StainAlive™ SSEA-4 antibody (DyLight™ 550) (Stemgent). After washing 3x for 5 min with 0.5 ml wash buffer, iPSCs stained with unlabelled primary antibodies were incubated with fluorescently labelled secondary antibodies, sheep anti-mouse FITC-labelled IgG (whole molecule) antibody (Sigma-Aldrich Chemie GmbH), and Cy3-labelled goat anti-rabbit IgG cross-adsorbed secondary antibody (Thermo Fisher Scientific) in 500 *μ*l of 1% BSA/wash buffer and incubated for 1-2 h at RT in the dark. Then, the cells were washed twice with 0.5 ml wash buffer and rinsed with 0.5 ml DPBS. After mounting the glass slide with a coverslip and Fluoroshield mounting medium containing DAPI (Abcam, Cambridge, UK), fluorescence images were taken using an Axiovert135 microscope and AxioVision 4.8.2 software (Carl Zeiss, Oberkochen, Germany).

### 2.8. Gene Expression Analysis

To analyze the quantitative expression of pluripotency genes and the presence of remaining srRNA, 1 × 10^6^ iPSCs were cultivated in 6-well plates until reaching 80-90% confluency. Total RNA was isolated using the Aurum™ Total RNA Mini Kit (Bio-Rad, Munich, Germany). For the qRT-PCR analysis, 300 ng RNA was reverse transcribed into complementary DNA (cDNA) using the iScript Kit (Bio-Rad) according to the manufacturer's instructions. Primer pairs were obtained from Ella Biotech GmbH (Martinsried, Germany) and used at a final concentration of 300 nM. Examined genes as well as used primer sequences are shown in Table 1. Real-time qRT-PCR reactions were run in a CFX Connect Real-Time PCR Detection System with the iQ™ SYBR® Green Supermix (Bio-Rad). PCR amplification of cDNA was performed under the following conditions: 10 min at 95°C for one cycle, followed by 40 cycles of 95°C for 15 s and 60°C for 60 s. All PCR reactions were performed in triplicates with a total volume of 15 *μ*l/well. Gene expression was normalized to human glyceraldehyde-3-phosphate dehydrogenase (GAPDH).

### 2.9. Trilineage Differentiation of iPSCs

To demonstrate pluripotency, iPSCs (passages 4 to 10) were differentiated using the StemMACS™ Trilineage Differentiation Kit (Miltenyi Biotec, Bergisch Gladbach, Germany) according to the manufacturer's instructions into the three embryonal germ layers: ectoderm, mesoderm, and ectoderm. Therefore, iPSCs were seeded with different cell numbers in VTN-N-coated wells of a 12-well plate: 1 × 10^5^ iPSCs for mesoderm differentiation, 2 × 10^5^ iPSCs for endoderm differentiation, and 1.5 × 10^5^ iPSCs for ectoderm differentiation. After 7 days of differentiation, cells were washed with DPBS and detached with 0.04% trypsin/0.03% EDTA and TNS (PromoCell) and centrifuged at 400 x g for 5 min. Afterwards, cells were washed with DPBS and fixated for 10 min at RT in FC Fixation Buffer (R&D Systems, Minneapolis, USA). After washing with DPBS, cells were suspended in wash buffer and stained with 5 *μ*l germ layer-specific fluorescence antibodies in 200 *μ*l of the cell suspension. Mesoderm differentiation was detected using PE-labelled mouse anti-human CD31 antibody (BD Biosciences, Franklin Lakes, USA) and Alexa Fluor 488-labelled anti-human *α*-smooth muscle actin (*α*-SMA) antibody (R&D Systems). PE-labelled anti-human *α*-fetoprotein (AFP) antibody (R&D Systems) and PE-labelled anti-human C-X-C chemokine receptor type 4 (CXCR4) antibody (R&D Systems) were used to detect endodermal differentiation. Ectodermal differentiation was demonstrated using PE-labelled anti-human paired box gene 6 (Pax6) antibody (Miltenyi Biotec) and Alexa Fluor 488-labelled anti-human neuron-specific class III *β*-tubulin (Tuj1) antibody (BD Biosciences). After incubation for 45 min at RT, cells were washed with 500 *μ*l wash buffer and suspended in 200 *μ*l CellFIX (BD Biosciences).

### 2.10. Teratoma Formation of iPSCs Using Chicken Embryo Chorioallantoic Membrane (CAM) Assay

The in vivo formation of teratomas and the trilineage differentiation potential of iPSCs were further investigated using the CAM assay. We followed the adapted protocol previously described by Steinle et al. [27]. After a 7-day incubation of the fertilized chicken embryos at 37°C, 2 × 10^6^ iPSCs were suspended in 50 *μ*l E8 medium containing 10 *μ*M Y-27632 ROCK inhibitor, mixed with 50 *μ*l Matrigel® (hECS qualified, Corning) and the suspension was carefully applied onto the CAM. Then, the eggs were sealed and further incubated for 10 days. At day 17, the cell aggregates on CAMs were excised around the application area and fixed with 4% paraformaldehyde (Merck, Darmstadt, Germany) overnight at 4°C. The specimens were washed with water, dehydrated with an ascending ethanol series, and embedded in paraffin for sectioning at 8 *μ*m thickness. Sections were stained with hematoxylin and eosin (H&E, Morphisto GmbH, Frankfurt, Germany).

### 2.11. Genomic Stability

The genomic stability of mRNA- and srRNA-derived iPSCs was analyzed by karyotyping. Therefore, fibroblasts and RNA-derived iPSCs (passages 5 to 12) were cultivated to reach about 50% confluency. Then, the cells were treated for 1 h with colcemid (Merck), incubated with 0.075 M KCl for 30 min at 37°C, and harvested in fresh fixative containing 25% acetic acid and 75% methanol. Karyotyping was performed on G-banded metaphase chromosomes using standard cytogenetic procedures. After GTG banding, about 15 metaphases were counted and 5 of them were structurally evaluated by G banding (banding quality of 400-500 bp).

### 2.12. Statistical Analysis

Data are shown as mean+standard deviation (SD) or standard error of the mean (SEM). Paired *t*-test or one-way analysis of variance (ANOVA) for repeated measurements followed by Bonferroni's multiple comparison test was performed to compare the means. Statistical analyses were performed double tailed using GraphPad Prism 6.01 (GraphPad Software, La Jolla, CA, USA). Differences of *p* < 0.05 were considered significant.

## 3. Results

### 3.1. RNA Synthesis

The first step for the successful reprograming of cells is the production of high-quality synthetic RNA molecules encoding the reprogramming factors. To perform the mRNA-based reprogramming, synthetic modified mRNAs containing each gene of interest (GOI) and UTRs were produced (Figure 1(a)). The mRNAs were generated using the modified nucleotides 5mCTP and Pseudo-UTP to completely replace cytosine and uridine. Furthermore, a 5′-cap structure (ARCA) and a 3′-end poly(A_120_) tail were added. After purification of the PCR product and the IVT, the products were analyzed using agarose gel electrophoresis to determine the specific length and purity. The detected bands showed the expected lengths of DNA templates and mRNAs (Klf4: 1.6 kb; cMyc: 1.5 kb; Oct4: 1.3 kb; Sox2: 1.1 kb; Lin28: 0.8 kb; and GFP: 0.9 kb).

An IRES-GFP encoding sequence was inserted into the plasmid containing nsP1 to nsP4 sequences of the VEE virus and the reprogramming factors [22] to enable the verification of transfection efficiency and the successful translation of the transfected srRNA in the cells during the reprogramming procedure (Figure 1(b)). After the linearization of the plasmid and the generation of srRNA, agarose gel electrophoresis was performed and bands at the expected length of about 17.7 kb were detected.

### 3.2. srRNA-Based Reprogramming Results in More Efficient and Straightforward iPSC Generation Compared to mRNA-Based Reprogramming

**Figure 2:**
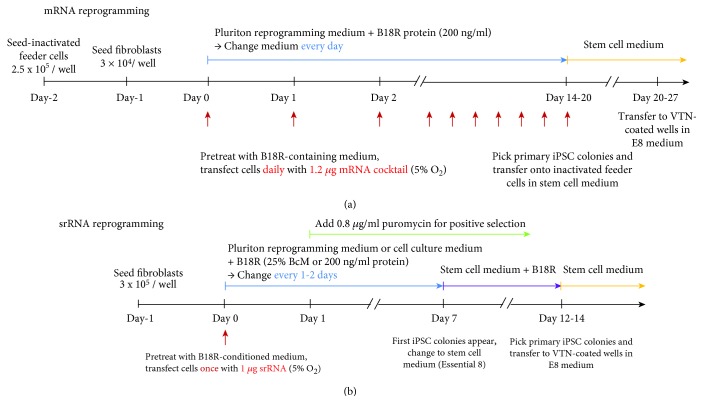
RNA-based iPSC reprogramming of fibroblasts: srRNA vs. mRNA. Timeline of (a) mRNA- or (b) srRNA-mediated reprogramming.

The reprogramming of fibroblasts with synthetic mRNAs required the daily transfection of the cells to maintain a constant level of reprogramming factor expression over 1 to 2 weeks. Thus, the repeated transfection of cells can cause stress and harm the cell viability, which in turn can decrease the reprogramming efficiency. Using the srRNA-based reprogramming, only one transfection was sufficient to maintain the expression level of reprogramming factors to generate iPSCs. The schedules for performing the reprogramming of cells with synthetic mRNA or srRNA are shown in Figure 2. Both RNA-based reprogramming approaches were performed at hypoxic conditions (5% O_2_) to improve efficiency [28].

**Figure 3:**
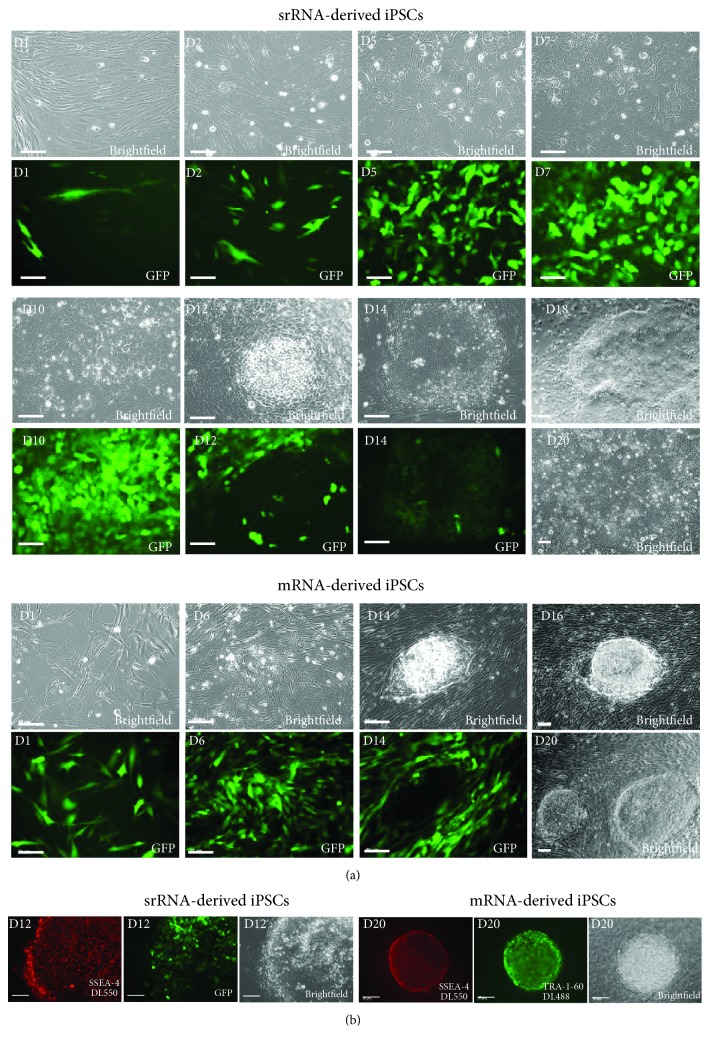
RNA-based iPSC reprogramming of fibroblasts: srRNA vs. mRNA. (a) Emerging of iPSCs over time and detection of GFP-expressing cells during the reprogramming period. Phase contrast and fluorescence microscopic pictures are shown. (b) Live-antibody staining of obtained iPSC colonies. The iPSCs obtained after 20 days of reprogramming by mRNAs were positively stained with DL550 SSEA-4 and DL488 TRA-1-60 antibodies. iPSCs obtained after 12 days of reprogramming by srRNA were positive for SSEA-4 and showed only partial GFP expression within the iPSC colony. Scale bars represent 100 *μ*m.

Already one day (D1) after the transfection of fibroblasts with the mRNA cocktail, which contained besides the reprogramming factor-encoding mRNAs also eGFP mRNA, a strong eGFP expression was detected (Figure 3(a)), which indicated a rapid translation of synthetic mRNA and an efficient delivery of mRNA into the cells. After 24 h, approximately 65% of the cells were eGFP positive and after the second transfection, about 90% of the cells expressed eGFP. The high eGFP expression could be sustained constantly over the period of the daily transfections (D1-D14). The first primary iPSC colonies appeared after 14-19 days, and the treatment with B18R protein was discontinued. After 14 days (D14), the cell morphology changed to an embryonic stem cell-like cell type and the eGFP expression in reprogrammed cells was diminished in tightly packed colonies, while the surrounding fibroblasts still strongly expressed eGFP. In the following days, iPSC colonies were expanded in the stem cell medium (D16, D19) without B18R to generate stable colonies. Live cell staining was performed with DL550-labelled SSEA-4 and DL488-labelled TRA-1-60 antibodies, 3 days after the last transfection. Cells exhibiting both markers were then picked for further cultivation on inactivated MEF feeder cells to support iPSC growth (Figure 3(b)). After 19-21 days of reprogramming, 20-25 iPSC colonies per well were obtained when 3 × 10^4^ fibroblasts were seeded per well of a 6-well plate (on 2.5 × 10^5^ inactivated NuFF feeders), which corresponds to a reprogramming efficiency of 0.8%.

Since in the synthetic mRNA-based reprogramming approach, the eGFP mRNA was cotransfected alongside with the reprogramming factor-encoding mRNAs, it can be not ensured that all cells are transfected with the same amount of eGFP mRNA or reprogramming factor-encoding mRNAs. In contrast, the GFP expression in srRNA-based reprogramming is directly comparable with the transfection efficiency, since all exogenously delivered RNA sequences are located on the same RNA construct.

Fluorescence microscopy revealed that 1 day (D1) after the transfection of 3 × 10^5^ cells with srRNA, only a few (1-3%) of the seeded cells were mostly expressing low levels of GFP. At the second day posttransfection (D2), the number of GFP-expressing cells increased to approximately 15% (Figure 3(a)). The increase of GFP-positive cells could be explained due to cell division and transfer of RNA to daughter cells. Furthermore, the delivered srRNA amount in the cells could be increased, which can lead to the detection of GFP in previously seemingly negative cells. After reaching confluency (D2-D3), puromycin was added for 3-7 days to the medium for positive selection of srRNA-containing cells. In general, after 2-3 days of puromycin treatment, all cells without srRNA died and only GFP-expressing cells were visible (Figure 3(a), D5). After 7 days of transfection, the morphology of most of the fibroblasts changed to an epithelial-like cell shape and the medium was changed to a stem cell medium (E8) containing 25% BcM. The daily microscopic monitoring of GFP expression in the cells showed that the change of the B18R-containing medium every two days is sufficient to maintain the srRNA in the cells. A daily medium change was performed when an increased number of dead cells were observed, e.g., after the puromycin treatment. The first colonies with typical iPSC morphology and positive SSEA-4 staining were obtained after 12 days of reprogramming (D12) (Figures 3(a) and 3(b)), while little or no GFP expression was detected in densely packed reprogrammed cells. Further cultivation and withdrawal of B18R resulted in the emergence of multiple iPSC colonies, which grew together until days 19 to 20 (D19-20) and covered most of the well surface. Thus, after 20 days of treatment, compared to synthetic mRNA-based reprogramming more iPSCs could be obtained after a single transfection with srRNA (Figure 3(a), D20). However, exact reprogramming efficiency cannot be determined, since at that time point, it cannot be distinguished whether the iPSC colonies are derived from a single parenteral cell or from their daughter cells.

### 3.3. Expression of Pluripotent Stem Cell-Specific Markers

**Figure 4:**
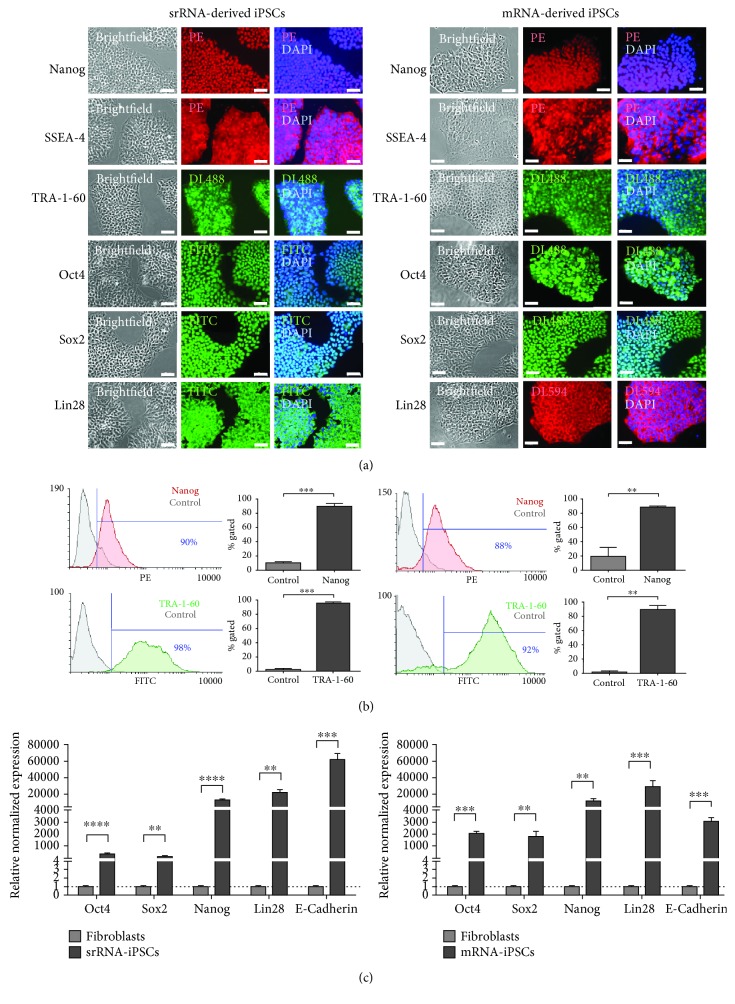
Analysis of pluripotency markers in iPSCs generated by mRNA or srRNA delivery. (a) iPSCs stained with antibodies specific for pluripotent stem cell markers (Nanog, SSEA-4, TRA-1-60, Oct4, Sox2, and Lin28) showed a strong protein expression (*n* = 3). Scale bars represent 50 *μ*m. (b) Analysis of Nanog and TRA-1-60 expression by flow cytometry. Data are shown as mean+SD (*n* = 3). Statistical differences were determined using a paired *t*-test (^∗∗^*p* < 0.01, ^∗∗∗^*p* < 0.001). (c) Analysis of Oct4, Sox2, Nanog, Lin28, and E-cadherin expression in iPSCs using qRT-PCR. mRNA levels were normalized to GAPDH mRNA levels, and the expression is presented relative to the expression levels in fibroblasts. Data are shown as mean+SEM (*n* = 3). Statistical differences were determined using a paired *t*-test (^∗∗^*p* < 0.01, ^∗∗∗^*p* < 0.001, and ^∗∗∗∗^*p* < 0.0001).

The expression of pluripotent stem cell-specific markers was analyzed using specific antibodies and fluorescence microscopy. iPSCs obtained by both srRNA- and mRNA-based reprogramming showed a strong expression of Nanog, Oct4, SSEA-4, TRA-1-60, and Lin28 (Figure 4(a)). Additionally, Nanog and TRA-1-60 expression in iPSCs was analyzed by flow cytometry (Figure 4(b)). Both proteins were highly expressed in almost all iPSCs generated by mRNA- or srRNA-based reprogramming. In srRNA-iPSCs, 90 ± 4% of the cells expressed Nanog and 98 ± 1% of the cells were positive for TRA-1-60. In mRNA-iPSCs, 88 ± 3% of the cells were Nanog positive and 92 ± 5% of cells expressed TRA-1-60. Furthermore, the expression of Oct4, Sox2, Nanog, Lin28, and E-cadherin was analyzed using qRT-PCR (Figure 4(c)). A significantly higher expression of Oct4, Sox2, Nanog, Lin28, and E-cadherin was detected in iPSCs generated by mRNA as well as srRNA compared to initial fibroblasts. However, the expression of Oct4 and Sox2 in mRNA-iPSCs was higher compared to srRNA-iPSCs (Oct4: 1956-fold versus 341-fold, Sox2: 1727-fold versus 163-fold). In contrast, E-cadherin expression in srRNA-iPSCs was higher than in mRNA-iPSCs (62453-fold versus 2964-fold). The expression of Nanog and Lin28 in srRNA-iPSCs was similar to the expression in mRNA-iPSCs.

### 3.4. In Vitro and In Vivo Differentiation Potential of Obtained iPSCs

#### 3.4.1. In Vitro Trilineage Differentiation

**Figure 5:**
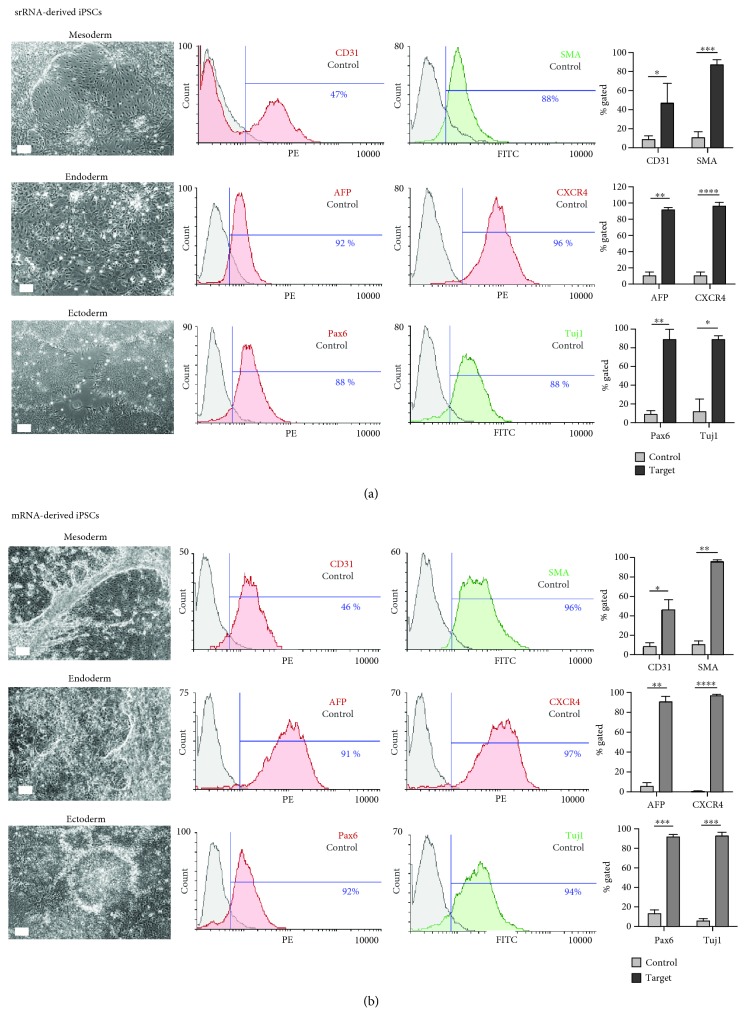
In vitro differentiation capacity of mRNA- or srRNA-iPSCs into the three germ layers: mesoderm, endoderm, and ectoderm. Microscopic images were taken 4 to 5 days after the differentiation of iPSCs, and cells with different morphologies were obtained depending on specific lineage differentiation. After 7 days of differentiation, flow cytometry analyses were performed with two specific antibodies for each lineage (mesoderm: CD31 and *α-*SMA; endoderm: AFP and CXCR4; ectoderm: Pax6 and Tuj1) and compared to the untreated control. Scale bars represent 100 *μ*m. The results are shown as mean+SD (*n* = 3). Statistical differences were determined using a paired *t*-test (^∗^*p* < 0.05, ^∗∗^*p* < 0.01, ^∗∗∗^*p* < 0.001, and ^∗∗∗∗^*p* < 0.0001).

To analyze the ability of iPSCs to differentiate into all three primary germ layers, mesoderm, endoderm and ectoderm, a directed 7-day differentiation protocol was performed. The obtained cells were examined by specific antibody staining and flow cytometry. After 4 to 5 days of differentiation, cells exhibiting the typical morphological structures of the mesodermal, endodermal, and ectodermal lineages were detected (Figure 5). The mesoderm induction led to the generation of elongated endothelial-like cells as well as smooth muscle-like cells. The endoderm differentiation resulted in the detection of cells similar to early hepatocyte-like cells. Cells arranged in neural rosettes were detected after the endoderm induction of iPSCs. Flow cytometric analysis of the cells obtained from srRNA-iPSCs (Figure 5(a)) revealed that 47 ± 20% of the cells were CD31 positive and 88 ± 5% SMA positive (mesoderm), 92 ± 3% were AFP positive and 96 ± 4% CXCR4 positive (endoderm), and 88 ± 11% of the cells were Pax6 positive and 88 ± 4% tubulin (Tuj1) positive (ectoderm). Cells derived from mRNA-iPSCs (Figure 5(b)) showed comparable results as srRNA-iPSC-derived cells: 46 ± 11% of the cells were CD31 positive and 96 ± 2% SMA positive (mesoderm), 91 ± 5% were AFP positive and 97 ± 1% CXCR4 positive (endoderm), and 92 ± 2% of the cells were Pax6 positive and 94 ± 3% Tuj1 positive (ectoderm).

#### 3.4.2. In Vivo Teratoma Formation

**Figure 6:**
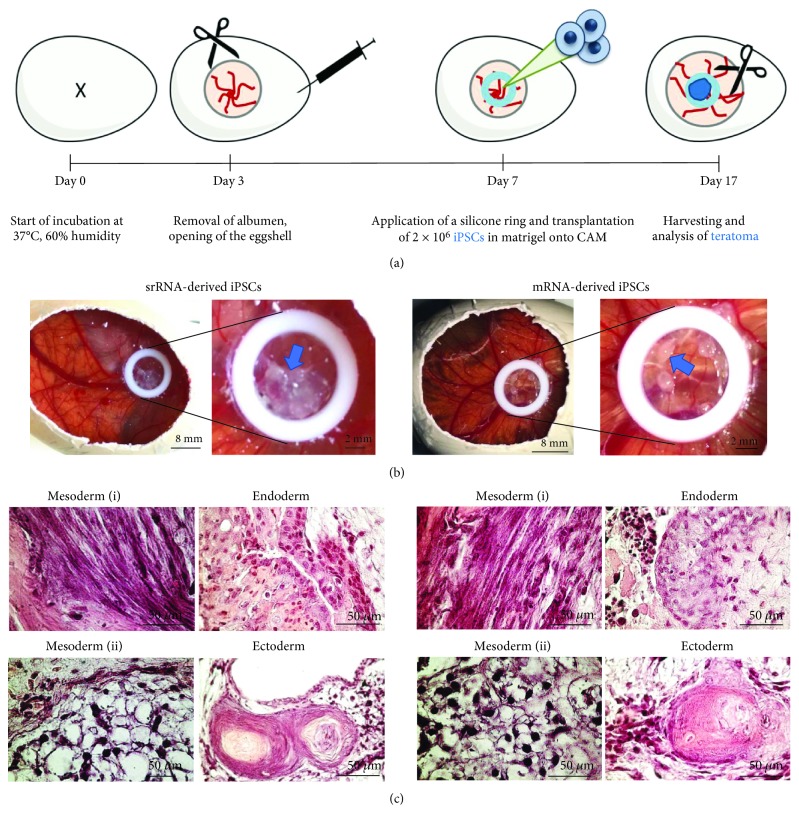
Analysis of teratoma formation after the application of mRNA- or srRNA-iPSCs onto the chicken embryo chorioallantoic membrane (CAM). (a) Schematic representation of the teratoma formation analysis on CAM. On day 7 of incubation, 2 × 10^6^ iPSCs were applied into the inner area of a silicone ring placed on CAM. (b) After 10 days (day 17), teratoma were formed on the CAM (indicated by arrows), excised, and embedded in paraffin. (c) Representative microscopic pictures of H&E-stained teratoma sections show the in vivo differentiation of iPSCs into cells of all three germ layers: mesoderm (i: striated muscle fibers, ii: adipocyte tissue), endoderm (glandular epithelium), and ectoderm (squamous epithelium).

The potential of iPSCs to differentiate into cell types of all the three germ layers was further analyzed in vivo. Therefore, iPSCs were applied 7 days after the incubation of fertilized eggs onto the CAM of chicken embryos (Figure 6(a)). After 10 days, small tumor-like cell masses were formed within the application area (silicone ring) as shown in Figure 6(b). H&E staining was performed with sections of 7 mm to detect the generated tissue structures. The mesodermal differentiation was confirmed by the formation of striated muscle fibers and adipocyte tissue (Figure 6(c)). Endodermal differentiation was demonstrated by the generation of the glandular epithelium and the ectodermal lineage differentiation was shown by the presence of the squamous epithelium.

### 3.5. Analysis of Genomic Abnormalities and Presence of srRNA Residues in srRNA-iPSCs

**Figure 7:**
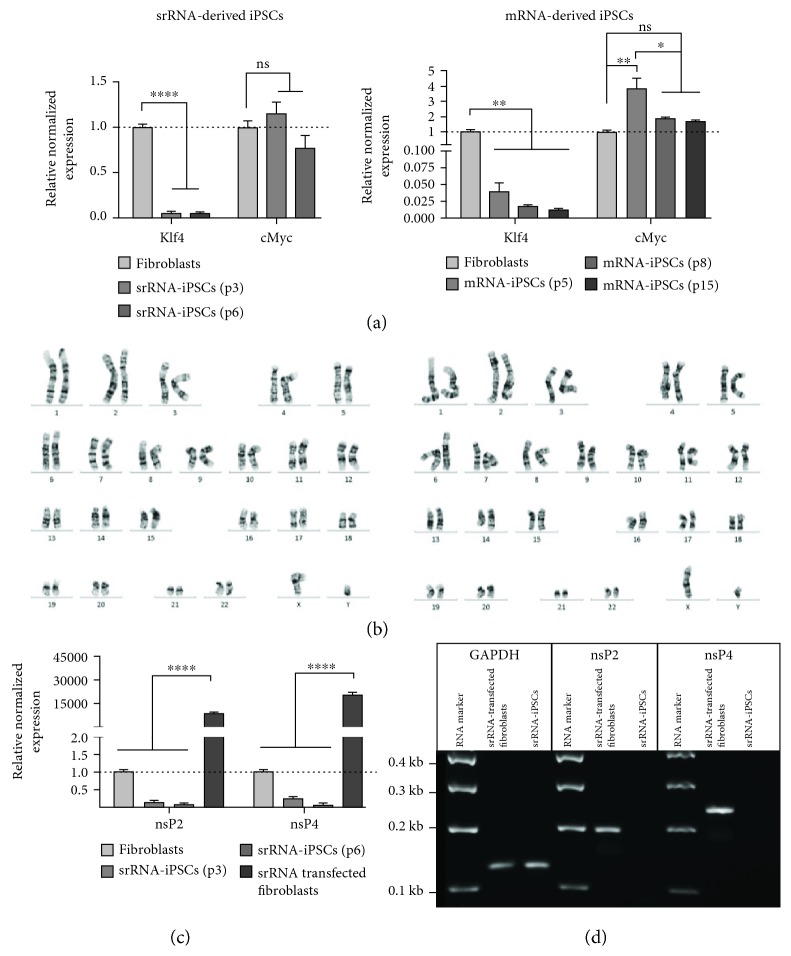
Analysis of genomic abnormalities, expression of prooncogenic factors, and the presence of srRNA residues in srRNA-iPSCs. (a) Detection of Klf4 and cMyc oncogene expression in iPSCs generated by srRNA and mRNA delivery using qRT-PCR. Results are shown as mean+SEM (*n* = 3). (b) Representative karyograms of iPSCs generated using srRNA or mRNA. (c) Detection of residual srRNA in obtained srRNA-iPSCs by performing qRT-PCR using nsP2- and nsP4-specific primers. Results are shown as mean+SEM (*n* = 3). (d) Analysis of specific PCR product lengths using 1% agarose gel electrophoresis. nsP2: 192 bases; nsP4: 238 bases; GAPDH: 126 bases. Statistical differences were determined using one-way ANOVA followed by Bonferroni's multiple comparison test (^∗^*p* < 0.05, ^∗∗^*p* < 0.01, and ^∗∗∗∗^*p* < 0.0001).

To examine possible genetic alterations in the iPSCs due to the reprogramming procedure, karyotyping was performed. The continued elevated expression of the oncogenes Klf4 and cMyc are associated with an increased tumorigenesis [29]; therefore, the expression of Klf4 and cMyc was also determined after the reprogramming using qRT-PCR. As shown in Figure 7(a), Klf4 expression was even significantly decreased in srRNA- and mRNA-iPSCs (different passages) compared to initial fibroblasts. In srRNA-iPSCs, already at passage 3, the expression of cMyc was not significantly different from the expression in the initial fibroblasts. However, in mRNA-iPSCs, the expression of cMyc was still elevated in the 5th passage, but decreased after further cultivation (passages 8 and 15) to the levels as in the initial fibroblasts.

The genetic stability/integrity of iPSCs was tested by the karyotyping of iPSCs and the initial fibroblasts. No changes regarding morphological structure such as size, centromere position, and band patterning were determined after the reprogramming procedure (Figure 7(b)). Both srRNA- and mRNA-iPSCs showed a normal male karyotype (46, XY), free of any discernible abnormalities.

After the appearance of the first srRNA-iPSC colonies, B18R was withdrawn from the medium to eliminate the reprogramming srRNA from the cells. To prove that the iPSCs are not containing residual srRNA, qRT-PCR analyses were performed using nsP1- and nsP4-specific primers. As a positive control, fibroblasts were transfected with srRNA; after 48 h, both the presence of nsP2 and the presence of nsP4 were demonstrated (Figure 7(c)). As expected, a 8826-fold higher nsP2 expression and a 20318-fold higher nsP4 expression were detected in srRNA-transfected fibroblasts, compared to those in untransfected fibroblasts. In srRNA-iPSCs (passages 3 and 6), no residual srRNA expression was measured compared to fibroblasts. In addition, PCR products were analyzed using 1% agarose gel electrophoresis (Figure 7(d)) and amplicons with expected lengths for GAPDH (126 bases), nsP2 (192 bases), and nsP4 (238 bases) were detected. Solely in positive control samples, nsP2- and nsP4-specific PCR products were visible.

## 4. Discussion

In recent years, the generation of patient-specific iPSCs from adult somatic cells has become a powerful tool in the field of tissue engineering and disease modeling and has led to great advances in regenerative medicine applications. In this study, we compared synthetic mRNA- and srRNA-based methods to generate footprint-free iPSCs from human fibroblasts regarding transfection and reprogramming efficiency, as well as overall workload and costs. Therefore, fibroblasts were reprogrammed either by multiple daily transfections with an mRNA cocktail consisting of 5 different reprogramming factor-encoding mRNAs and an eGFP mRNA [6], or by a single transfection with srRNA, which enables the sustained expression of reprogramming factors [22]. To monitor the transfection efficiency and the translation of srRNA, an additional sequence encoding an IRES [30] and GFP was added to allow the cap-independent initiation of translation. During the reprogramming process, the treatment of cells with B18R suppresses the cellular type I interferon immune response to the srRNA [31] and prevents the premature degradation of the srRNA. After the reprogramming of cells, the withdrawal of B18R from the medium leads to the degradation and elimination of srRNA [22].

The obtained iPSCs showed the expression of typical pluripotency markers and the potential to differentiate into the cells of the three germ layers in vitro and in vivo. The generated iPSCs showed no genomic abnormalities, and no residual srRNA could be found in the iPSCs generated by srRNA. However, the comparison of both methods clearly revealed that the srRNA-based reprogramming is more efficient and convenient than the synthetic mRNA-based method (Table 2). The costs for the synthesis and purification of one microgram of mRNA or srRNA are comparable (approximately 2.5€/1 *μ*g). A key advantage of this method is the about 24 times lower production costs due to the one-shot transfection of cells with 1 *μ*g srRNA (2.5€) compared to the required daily transfection of cells with 1.2 *μ*g mRNA cocktail containing 6 different nucleoside-modified mRNAs for about 20 days (60€). Furthermore, using the srRNA, iPSCs were obtained earlier than after the mRNA transfection. The srRNA also contains an open reading frame for puromycin resistance to enable the positive selection of srRNA-containing cells. Due to the positive selection with puromycin during the early time point of reprogramming, only cells containing the srRNA could survive; therefore, the reprogramming efficiency was increased.

In this work, an IRES-GFP sequence was added to the reprogramming factor-encoding srRNA, which allowed the direct control of successful transfection and translation of the srRNA during the reprogramming of cells. Additionally, the absence of B18R in the medium led to the decrease of fluorescence intensity, which indicated the degradation of srRNA. Through daily monitoring of GFP expression, we were able to adjust the medium replacement schedule of the B18R-containing medium to a 2-day rhythm. In the case of the synthetic mRNA-based application, the eGFP expression was also used to monitor the transfection efficiency and the translation of synthetic mRNAs in the cells. Therefore, the mRNA cocktail contained eGFP-encoding mRNA, which was simultaneously transfected into the cells with the reprogramming factor-encoding mRNAs. But, compared to the srRNA-containing GFP encoding sequence, the monitoring of synthetic mRNA-transfected cells was less precise, since the delivery of each single mRNA amount can differ in each cell and the consistent supply of all 6 individual mRNAs into the same cells cannot be ensured every day in the same manner. Furthermore, prior to starting with the reprogramming, the transfectability of somatic cells can be analyzed using the srRNA-containing GFP encoding sequence. Thereby, the required transfection reagent and the duration of transfection can be determined for different types of cells.

The use of RNA-based molecules for the expression of transcription factors in the cells is integration free. The delivery of synthetic mRNA into the cells leads to the transient expression of desired proteins for commonly about 2-3 days in the cells [6, 9, 10]. After the uptake of srRNA into the cells, the expression of nonstructural proteins (nsP1-nsP4) enables repeated replication of RNA in the cytosol and thereby a prolonged protein expression. The degradation of srRNA in the obtained iPSCs can be proven by qRT-PCR using nsP2- and nsP4-specific primers. In our studies, in early passage iPSCs (passages 3-5), no residual srRNA could be detected after the reprogramming. Furthermore, the decrease of cMyc and Klf4 expression also indicates the degradation of the srRNA construct in the iPSCs. These proteins are required during the reprogramming procedure; however, afterwards, their expression should be downregulated since the permanent overexpression of these proteins is linked with an increased tumorigenesis [29, 32] and can be found in different types of cancer [33, 34].

To reprogram somatic cells with synthetic RNAs, the use of interferon inhibitor B18R is required [6, 21, 22]. Thus, the medium can be supplemented either with 200 *μ*g/ml recombinant B18R protein or a conditioned medium containing B18R (BcM) can be used. The use of BcM instead of B18R recombinant protein reduces the costs, and the conditioned medium can further provide additional proteins, e.g., fibroblast growth factors (FGF-2), which can support the reprogramming procedure. This can be beneficial when serum-free medium, e.g., E8 stem cell medium, is used. The ratio of BcM to culture or reprogramming medium is adjustable. In this study, medium containing fresh 25% BcM resulted in successful reprogramming, but the BcM amount can be increased to, for example, 50%, if a weak GFP signal is detected in transfected cells.

In this study, for a better comparability with the mRNA reprogramming approach, Pluriton reprogramming medium was used during the srRNA-based reprogramming. However, this is not explicitly required for successful and efficient reprogramming with srRNA. Yoshioka et al. generated iPSCs by using fibroblast culture medium instead of Pluriton [22]. Furthermore, it is also possible to substitute the only animal component FBS in the cell culture medium with human serum or platelet lysate, to generate iPSCs under xeno-free conditions from different cell types [35].

In this work, the reprogramming of newborn human fibroblasts was performed. However, Yoshioka and Dowdy also successfully generated iPSCs from adult human fibroblasts of 54- to 77-year-old healthy donors and from a 24-year-old cardiomyopathy patient using srRNA encoding the reprogramming factors Oct4, Sox2, Klf4, Glis1, and cMyc [24]. In our recent study, we could also demonstrate the successful reprogramming of human adult jaw periosteal cells into iPSCs using srRNA encoding Oct4, Klf4, Sox2, and cMyc [35]. These studies demonstrated that also adult somatic cells can be reprogrammed using srRNA. Another interesting source of adult cells for reprogramming are blood-derived cells, such as peripheral blood mononuclear cells (PBMCs) or endothelial progenitor cells (EPCs), which can be obtained by minimally invasive blood collection from healthy donors or patients. Poleganov et al. reported the successful reprogramming of human blood-outgrowth EPCs using an mRNA-based approach [36]. Therefore, the use of blood-derived cells to generate iPSCs by srRNA-based reprogramming would represent another promising cell source for adult cells.

In previous studies, a reprogramming efficiency of 4.4% was achieved after the reprogramming of BJ fibroblasts with synthetic modified mRNAs [6, 20]. In our studies, we obtained a reprogramming efficiency of 0.8% by applying the same protocol to NuFFs, this was probably caused by lab-to-lab or material variabilities. Furthermore, the use of adult fibroblasts or fibroblasts from diseased patients as well as the use of other somatic cells can result in other reprogramming efficiencies [6, 24, 37]. Therefore, reprogramming protocols should be tested and optimized for each cell type [7]. Moreover, the reprogramming efficiency of the mRNA-based approach depends on the initial cell density [20, 21], and it is promoted by high cell cycling rates [38]. By using mitotically arrested feeder cells, lower target cell counts can be used for initial seeding, thereby promoting the reprograming process. However, feeder-free protocols are beneficial to circumvent additional variabilities and the risk of contamination with xenogeneic material [20].

Recently, Kogut et al. established another RNA-based feeder-free protocol for the reprogramming of neonatal, adult, and senescent human fibroblasts [21]. Here, a different set of synthetic modified mRNAs encoding M3O (OCT4 fused with the MyoD transactivation domain), Sox2, Klf4, cMyc, Lin28A, Nanog, and miRNA-367/302s were applied. Using this method and only 500 primary cell neonatal fibroblasts, the reprogramming efficiency was highly increased and the RNA transfection was reduced to every 2 days with 0.6 *μ*g mRNA cocktail. These results would suggest that the reprogramming efficiency of the srRNA-based reprogramming method could be further improved by the addition of reprogramming enhancers/modulators, such as valproic acid, TGF-*β* inhibitors, vitamin C, butyrate, or miRNAs [7, 8, 21, 39–42].

## 5. Conclusions

The footprint-free iPSCs obtained by srRNA- and synthetic mRNA-based reprogramming are promising cells to generate desired cell types for clinical application. However, the single-shot application of srRNA allowed a more time- and cost-efficient generation of unlimited numbers of iPSCs without any genomic integration compared to the daily transfection of multiple reprogramming factor-encoding mRNAs. We believe that this method holds great promise for the integration-free reprogramming of any somatic cells, due the comfortable experimental setup with only one srRNA administration, direct GFP monitoring, and higher reprogramming efficiency. The highly efficient generation of footprint-free iPSCs and the efficient differentiation into desired cells will increase the potential of this technology in translational research, therapy, and disease modeling.

## Figures and Tables

**Table 1:** List of primer sequences used for qRT-PCR analysis.

Gene	Forward primer (5′ → 3′)	Reverse primer (5′ → 3′)
Pluripotency marker		
GAPDH	TCAACAGCGACACCCACTCC	TGAGGTCCACCACCCTGTTG
Oct4 [3]	AGCGAACCAGTATCGAGAAC	TTACAGAACCACACTCGGAC
Sox2 [3]	AGCTACAGCATGATGCAGGA	GGTCATGGAGTTGTACTGCA
Nanog [3]	TGAACCTCAGCTACAAACAG	TGGTGGTAGGAAGAGTAAAG
Lin28	CTTCTTCTCCGAACCAACC	CAGCCACCTGCAAACTG
E-cadherin	TATACCCTGGTGGTTCAAGC	CACCTGACCCTTGTACGTG
Klf4 [3]	TCTCAAGGCACACCTGCGAA	TAGTGCCTGGTCAGTTCATC
cMyc [3]	ACTCTGAGGAGGAACAAGAA	TGGAGACGTGGCACCTCTT
srRNA-specific marker		
nsP2	TCCACAAAAGCATCTCTCGCCG	TTTGCAACTGCTTCACCCACCC
nsP4	TTTTCAAGCCCCAAGGTCGCAG	TGTTCTGGATCGCTGAAGGCAC

GAPDH: glyceraldehyde-3-phosphate dehydrogenase; Oct4: octamer binding transcription factor 4; Sox2: sex-determining region Y-box 2; E-cadherin: epithelial cadherin; Klf4: Krüppel-like factor 4; cMyc: cellular myelocytomatosis; nsP: nonstructural protein.

**Table 2:** Advantages and disadvantages of srRNA- or mRNA-based reprogramming of fibroblasts to obtain footprint-free iPSCs.

	srRNA reprogramming	mRNA reprogramming
Advantages of srRNA		
RNA generation	No modified nucleotides^∗^	Modified nucleotides^∗^
	Identical RNA molecules	Multiple mRNAs
RNA transfection	Once (1 *μ*g)	Daily (1.2 *μ*g) for 14-20 days
Transfection efficiency	GFP reporter on the same srRNA construct	Additional transfection with GFP mRNA for monitoring
First iPSCs emerged after	12 days	14 days
Reprogramming efficiency	Very high efficiency after positive selection	High efficiency (0.8%)
Reprogramming costs of RNA^∗∗^	~2.5€ (1 *μ*g srRNA once)	~60€ (~2.5€/1 *μ*g mRNA for 20 days)
Transgene-free iPSCs	Yes (total elimination of srRNA was demonstrated after p3)	Yes (no integration of mRNA into the host genome)
Disadvantages of srRNA		
RNA modification	Posttranscriptional enzymatic 5′-capping and 3′-polyadenylation	Cotranscriptional 5′-capping and 3′-polyadenylation
Immune system activation counteraction	Interferon inhibitor B18R required^∗∗∗^	Interferon inhibitor B18R required^∗∗∗^
Transgene expression	Check for residual srRNA expression (VEE virus-derived RNA construct)	Natural degradation of mRNA in cells after 2-3 days

^∗^Modified nucleotides (e.g., 5mCTP, Pseudo-UTP, and N1-methylpseudo-UTP) can improve the translation of proteins but are also expensive to purchase. ^∗∗^Costs for the synthesis and purification of RNA (with commercially available kits, without plasmid generation) needed for one reprogramming experiment. ^∗∗∗^B18R-containing medium (BcM) can vary from batch to batch; therefore, the functionality of B18R has to be assessed before use, for example, by the determination of the positive transfection of fibroblasts with GFP mRNA with 25% BcM compared to the transfection without B18R. To date, there are no commercially available antibodies against B18R for specific analysis of the B18R content. For a constant quality, B18R can also be purchased as a recombinant protein, but this is much more expensive than the use of BcM.

## Data Availability

The data used to support the findings of this study are included within the article and are available from the corresponding author upon request.
